# The NIMA-related kinase family and cancer

**DOI:** 10.3389/fonc.2025.1556917

**Published:** 2025-03-27

**Authors:** Haotian Li, Junhan Li, Yue Zhang, Ran Cao, Congcong Guo, Mingwen Jiao

**Affiliations:** ^1^ Department of Endocrinology and Metabology, The First Affiliated Hospital of Shandong First Medical University and Shandong Provincial Qianfoshan Hospital, Shandong First Medical University, Shandong Key Laboratory of Rheumatic Disease and Translational Medicine, Shandong Institute of Nephrology, Jinan, China; ^2^ Jining Medical University, Jining, Shandong, China; ^3^ School of Clinical Medicine, Shandong Second Medical University, Weifang, China; ^4^ Department of General Surgery, The First Affiliated Hospital of Shandong First Medical University and Shandong Provincial Qianfoshan Hospital, Jinan, China

**Keywords:** NEK family, mitosis, therapeutic target, cancer, proliferation, metastasis

## Abstract

Cancer is a disease where cells begin to divide uncontrollably and spread to other parts of the body. Mitotic kinases play a crucial role in the initiation and progression of all human malignancies, making them common therapeutic targets. However, a significant portion of the human kinome has yet to be functionally studied in cancer systems. The NIMA-related kinase family (NEKs), consisting of 11 members distributed across different cellular regions, are important protein kinases that regulate mitotic processes. Emerging research suggests that NEK family members have potential key roles in various malignancies. This review systematically summarizes the expression and regulatory mechanisms of NEK family members in different cancer systems, highlighting that targeting NEKs holds promise as a new therapeutic approach for inhibiting cancer growth and metastasis.

## 1 Background

According to data from the World Health Organization, cancer is an important cause of death globally. It was reported that in 2022,there were 19,976,499 new cases of malignant tumors worldwide, an increase of nearly 700,000 compared to 2020 (1). Among these, the highest incidence of malignancies was in lung cancer, followed by breast cancer, colorectal cancer, prostate cancer, stomach cancer and liver cancer. The most challenging obstacles in cancer therapy is the gradual development of therapy resistance by patients, leading to tumor recurrence. Therefore, discovering new targets that can restore treatment sensitivity or provide alternative signal pathways for targeting is a key focus in therapeutic research. Kinases are commonly used targets in oncology (2).The human kinome is vast, comprising 518 kinases, offering a wealth of potential targets for oncology (3).Currently,253 kinase-specific inhibitors are in phases 1-4 of clinical trials (4).Inhibiting kinase activity and preventing the corresponding phosphorylation cascades affect various pathways of tumorigenesis, including cell proliferation,apoptosis, and cell migration. Despite the availability of several kinase-targeted therapies, many kinases within the human kinome remain underexplored, potentially holding new therapeutic targets.

**Figure 1:**
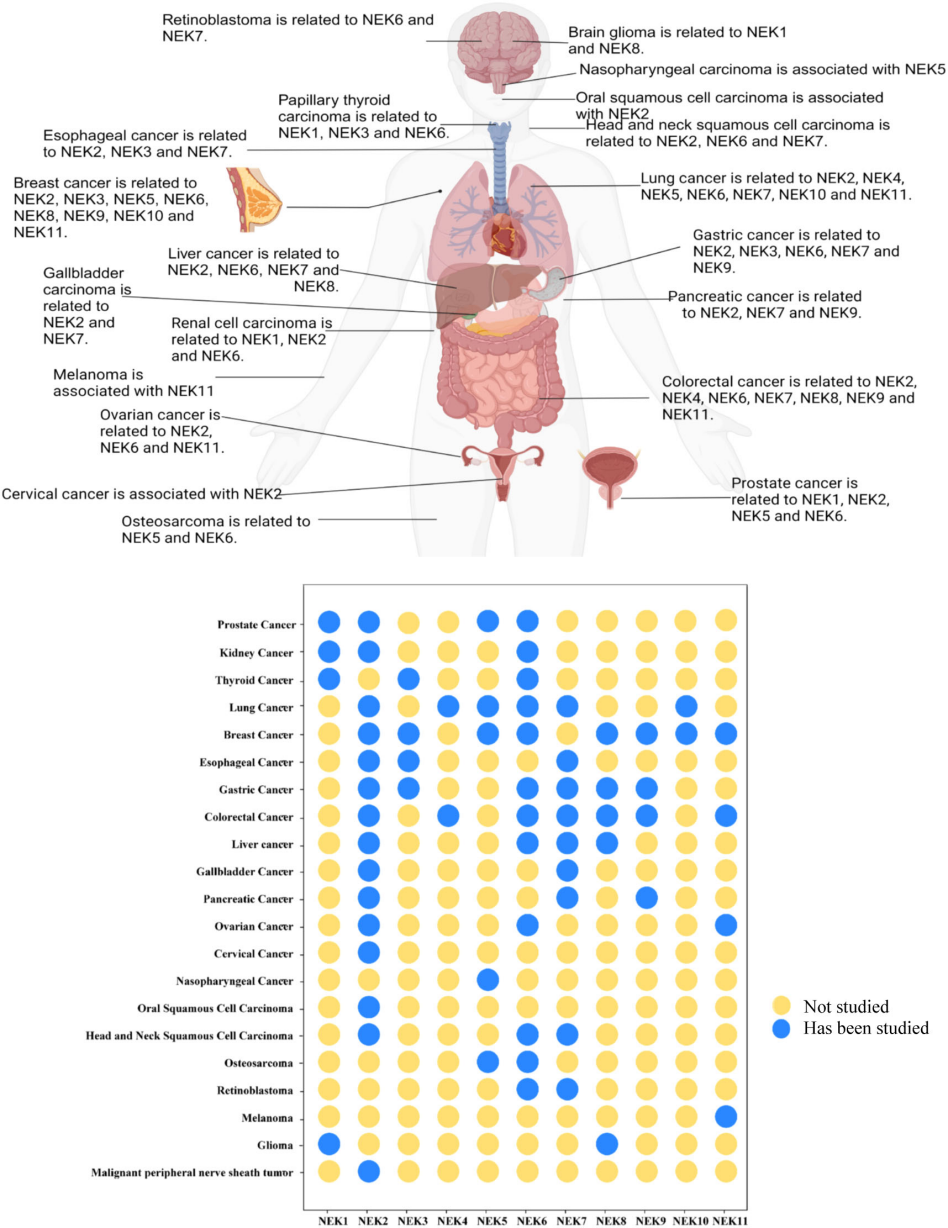
Summary of research progress on the NEK family in the occurrence and development of various cancers based on the literature.(Created in BioRender. Fengying, S. (2025) https://BioRender.com/c45s254).

The NIMA-related kinase (NEK) family is a group of serine/threonine protein kinases that play an important role in regulating mitotic processes. In addition to cyclin-dependent kinases (CDKs),Aurora kinases, and Polo-like kinases (PLKs), NEKs are among the most-known kinase families, initially recognized for their role in cell cycle regulation (5).Mammals are composed of 11 NEKs, respectively are NEK1, NEK2, NEK3, NEK4, NEK5, NEK6, NEK7, NEK8, NEK9, NEK10 and NEK11, which are distributed across cilia, centrosomes, the nucleus, cytoplasm, and mitochondria (6).Recent studies have increasingly shown that NEK family members are also involved in mRNA splicing, myogenic differentiation, inflammasome formation, protein transport within the cell, mitochondrial homeostasis, and DNA damage, with their abnormal expression or function closely related to tumor progression. They are associated with the occurrence and development of breast cancer,lung cancer,prostate cancer, pancreatic cancer, thyroid cancer, colorectal cancer, and hepatocellular carcinoma and are associated with poor prognosis for various cancers, making them potential therapeutic targets (7, 8). In recent years, information about the NEK family has been accumulating. Consider the versatility of the NEK family, this article outlines the research of the NEK family in various cancers (**Figure 1**), as well as the clinical research of the NEK family as a potential biomarker in oncology (**Table 1**). Meanwhile we introduce the molecular mechanism of the NEK family (**Table 2**) and the current summary of potential therapeutic inhibitors of the NEK family(**Table 3**), aiming to provide researchers with a fuller and more comprehensive understanding of the NEK family and to offer a foundation for exploring tumorigenesis mechanisms and designing anti-tumor drugs.

**Table 1:** In clinical research, the potential biomarkers for the NEK family in oncology.

Kinases	Cancer type	Sample Type	Biomark	Trend(vs control)	References
NEK1	Postate Cancer	Prostate cancer tissue	p-NEK1-T141**↑**	Rise	Vibha Singh et al.,2019 (19)
	Renal Cancer	RCC tissue	NEK1**↑**	Rise	Yumay Chen et al.,2014 (22)
	Glioma	Glioma tissue	NEK1**↑**	Rise	Jun Zhu et al.,2016 (21)
	Thyroid Cancer	Thyroid tumor tissue	NEK1**↑**	Rise	Yanbing Shen et al.,2022 (20)
NEK2	Prostate Cancer	Prostate cancer tissue	NEK2**↑**	Rise	F-B Zhang et al.,2019 (27)
	Renal Cancer	RCC tissue	NEK2**↑**	Rise	Xiaoli Feng et al.,2023 (28)
		RCC tissue	NEK2**↑**	Rise	Jiancheng Zhou et al.,2022 (126)
	Non-Small Cell Lung Cancer	Non-Small Cell Lung Cancer tissue	NEK2**↑**	Rise	Rui Bai et al.,2021 (127)
		Non-Small Cell Lung Cancer tissue	NEK2↑	Rise	Xinwen Zhong et al.,2014 (51)
	Lung Cancer	Lung cancer tissue	NEK2**↑**	Rise	Dejian Zhao et al.,2017 (31)
	Breast Cancer	Breast cancer tissue	NEK2**↑**	Rise	Zeyu Xing et al.,2021 (33)
		Breast cancer tissue	NEK2**↑**	Rise	Yuanwen Chen et al.,2020 (32)
		Breast cancer tissue	NEK2**↑**	Rise	Daniel G Hayward et al.,2004 (55)
	Gastric Cancer	Gastric cancer tissue	NEK2↑	Rise	Jianyong Wu et al.,2024 (35)
		Gastric cancer tissue	NEK2**↑**	Rise	Hao Wan et al.,2021 (34)
	Esophageal squamous cell carcinoma	Esophageal squamous cell carcinoma tissue	NEK2↑	Rise	Shaorui Gu et al.,2024 (36)
	Colon Cancer	Colon Cancer tissue	NEK2↑	Rise	Lei Lu et al.,2015 (36)
	Hepatocellular Carcinoma	Hepatocellular carcinoma tissue	NEK2↑	Rise	Mei-Xia Zhang et al.,2016 (42)
		Hepatocellular carcinoma tissue	NEK2**↑**	Rise	Shuang Lin et al.,2016 (41)
		Hepatocellular carcinoma tissue	NEK2**↑**	Rise	Yi Zhang et al.,2018 (58)
	Pancreatic Cancer	Pancreatic Cancer tissue	p-NEK2**-**194**↑**	Rise	Xiaozhen Zhang et al.,2021 (43)
		Pancreatic Cancer tissue	NEK2**↑**	Rise	Toshio Kokuryo et al.,2016 (57)
	Ovarian Cancer	Ovarian cancer tissue	NEK2**↑**	Rise	Xia Liu et al.,2014 (45)
		Ovarian cancer tissue	NEK2↑	Rise	X.-L. LIU et al.,2019 (46)
	Oral squamous cell carcinoma	Oral squamous cell carcinoma tissue	NEK2↑	Rise	Ting-Ting Ye et al.,2023 (49)
	Malignant peripheral nerve sheath tumor	Malignant peripheral nerve sheath tumor tissue	NEK2**↑**	Rise	Thomas P Stricker et al.,2013 (49)
NEK3	Breast Cancer	Breast cancer tissue	p-NEK3-T165**↑**	Rise	Katherine M. Harrington et al.,2016 (64)
	Gastric Cancer	Gastric cancer and adjacent tissue	NEK3**↑**	Rise	Yongfeng Cao, MD et al.,2017 (63)
	Thyroid Cancer	Thyroid tumor tissue	NEK3**↑**	Rise	Talita Diniz Melo-Hanchuk et al.,2020 (6)
NEK4	Lung Cancer	Lung cancer tissue	NEK4**↑**	Rise	So Jung Park et al., 2016 (68)
	Colorectal Cancer	Colorectal cancer tissue	NEK4**↑**	Rise	So Jung Park et al., 2016 (68)
NEK5	Breast Cancer	Breast cancer tissue	NEK5**↑**	Rise	Jing Pei et al., 2019 (128)
	Postate Cancer	Prostate cancer tissue	NEK5**↑**	Rise	Anastasia S Nikitina et al., 2017 (68)
	Nasopharyngeal carcinoma	Nasopharyngeal carcinoma tissue	NEK5**↑**	Rise	C-H Zhao et al., 2020 (78)
NEK6	Breast Cancer	Breast cancer tissue	NEK6**↑**	Rise	Wen-Liang Gao et al.,2022 (80)
		Breast cancer tissue	NEK6**↑**	Rise	Zhixian He et al.,2018 (81)
	Hepatocellular Carcinoma	Hepatocellular carcinoma tissue	NEK6**↑**	Rise	Hao Zhang et al.,2023 (83)
		Hepatocellular carcinoma tissue	NEK6**↑**	Rise	Xiaolei Cao et al.,2012 (82)
		Hepatocellular carcinoma tissue	NEK6**↑**	Rise	Biao Zhang et al.,2014 (84)
	Head and neck squamous cell carcinoma	Head and neck squamous cell carcinoma tissue	NEK6**↑**	Rise	Zhi-Min Yang et al.,2022 (87)
	Gastric Cancer	Gastric cancer tissue	NEK6**↑**	Rise	Ji Xu et al.,2015 (86)
	Ovarian Cancer	Ovarian cancer tissue	NEK6**↑**	Rise	Jingchun Liu et al., 2024 (129)
	Serous Ovarian cancer	Serous Ovarian cancer tissue	NEK6**↑**	Rise	Marta De Donato et al., 2015 (129)
	Osteosarcoma	Osteosarcoma tissue	NEK6**↑**	Rise	Min Zhu et al., 2023 (92)
	Colon Adenocarcinoma	Colon Adenocarcinoma tissue	NEK6**↑**	Rise	Zhongshi Hong et al.,2022 (93)
	Renal Cancer	Renal Cancer tissue	NEK6**↑**	Rise	Yifei Liu et al.,2022 (94)
	Thyroid Cancer	Thyroid tumor tissue	NEK6**↑**	Rise	Fukun Chen et al.,2018 (96)
		Thyroid tumor tissue	circ_NEK6 **↑**	Rise	Fukun Chen et al.,2021 (97)
NEK7	Squamous Cell Carcinoma of the Head and Neck	Head and neck cancer tissue	NEK7**↑**	Rise	Vassiliki Saloura et al.,2015 (98)
	Gallbladder cancer	Gallbladder cancer tissue	NEK7**↑**	Rise	R Wang et al.,2013 (99)
	Hepatocellular Carcinoma	HCC tissue	NEK7**↑**	Rise	Lei Zhou et al.,2016 (102)
		HCC tissue	NEK7**↑**	Rise	Zilong Yan et al.,2022 (101)
	Pancreatic Cancer	Pancreatic Cancer tissue	NEK7**↑**	Rise	Zilong Yan et al.,2021 (103)
	Esophageal Adenocarci-noma	Esophageal Adenocarci-noma tissue	NEK7**↑**	Rise	Lei Chen et al.,2023 (62)
	Gastric Cancer	Gastric cancer tissue	NEK7**↑**	Rise	Yi-Ke Li et al.,2021 (105)
NEK8	Breast Cancer	Breast cancer tissue	NEK8**↑**	Rise	Wen-Liang Gao et al.,2022 (80)
		Breast cancer tissue	NEK8**↑**	Rise	Alex J. Bowers et al.,2003 (130)
		Breast cancer tissue	NEK8**↑**	Rise	Eunji Kang et al.,2023 (131)
	Glioma	Glioma tissue	NEK8**↑**	Rise	Meng Xiao et al.,2021 (111)
	Hepatocellular Carcinoma	HCC tissue	NEK8**↑**	Rise	Wen-Ling Chan et al.,2013 (110)
	Colorectal Carcinoma	Colorectal Carcinoma tissues	NEK8**↑**	Rise	WANG Lu et al.,2017 (132)
		Colon Cancer tissue	NEK8**↑**	Rise	Ruifang Sun et al.,2023 (113)
NEK9	Breast Cancer	Breast cancer tissue	NEK9**↑**	Decline	ZIRU XU et al.,2019 (116)
		Breast cancer tissue	NEK9↑	Rise	Wen-Liang Gao et al.,2022 (80)
		Peripheral blood	NEK9**↑**	Rise	Zhu Zhaoyu et al.,2022 (115)
	Gastric Cancer	Gastric cancer tissue	NEK9**↑**	Rise	Guofang Lu et al.,2023 (117)
		Gastric cancer tissue	NEK9**↑**	Rise	Guofang Lu et al.,2021 (118)
	Colon Cancer	Colon Cancer tissue	NEK9**↑**	Rise	Meejeong Kim et al.,2023 (120)
	Pancreatic Cancer	Pancreatic Cancer tissue	NEK9**↑**	Rise	Huizhen Nie et al.,2021 (133)
NEK10	Breast Cancer	Breast cancer tissue	NEK10**↑**	Decline	Wen-Liang Gao et al.,2022 (80)
	Lung Adenocarcinoma	Lung Adenocarcinoma tissue	NEK10**↑**	Rise	Previn Dutt et al.,2024 (122)
NEK11	Melanoma	Blood Melanoma tissue	NEK11**↑**	Rise	Eirini Christodoulou et al.,2020 (124)
	Colorectal Cancer	Colorectal Cancer tissue	NEK11**↑**	Rise	Sarah R. Sabir et al.,2015 (123)
	Ovarian Cancer	Ovarian Cancer	NEK11↑	Decline	Xia Liu et al.,2014 (125)

**Table 2:** Details molecular mechanism of NIMA-related kinase family.

Kinases	Cancer type	Cell line	Animal	Main functions	Upstream/Downtream pathway	References
NEK1	Postate Cancer	LNCaP cells	NOD-SCID mice	Inhibition of Apoptosis	TLK1**↑** - NEK1**↑**	Vibha Singh et al.,2019 (19)
		LNCaP	TRAMP-NEK1+/− mice	Proliferation	TLK1B**↑** - NEK1**↑ -** pYAP1 - Y407**↑**	Ishita Ghosh et al.,2023 (18)
		TRAMP-C2	TRAMP mice	Proliferation	TLK1**↑**- Nek1 **↑**	Vibha Singh et al.,2019 (16)
		LNCaP, HeLa, NT1	TRAMP-NEK1+/− mice	Proliferation	TLK1**↑** - NEK1**↑** - YAP1**↑**	Md Imtiaz Khalil et al., 2020 (134)
	Renal cancer	Caki-1, ACHN,786-O, A-498 769-P		Apoptosis	VHL**↑** - HIF-2α**↓** - NEK1**↓**	Guang Chen et al., 2019 (23)
		A498、786-O		Apoptosis	NEK1**↓** - VDAC1**↓**	Yumay Chen et al., 2014 (22)
NEK2	Renal Cancer	A498, Caki-1, 786-O	BALB/c nude mice	Proliferation,Migration, Invasion	NEK2**↑** - Wnt/β-catenin**↑**	Jiancheng Zhou et al.,2022 (126)
	Non-Small Cell Lung Cancer	A549,PC9,H1299,H1975	nude mice	Proliferation,Migration, Invasion	NEK2**↑** - Wnt/β-catenin**↑**	Rui Bai et al.,2021 (127)
	Lung Cancer	SK-MES-1		Apoptosis	miRNA-128**↑** - NEK2**↓**	Dejian Zhao et al.,2017 (31)
	Breast Cancer	MCF-7		Inhibition of Proliferation,Migration, Invasion	miR-128-3p**↑** - NEK2**↓ -** Wnt/β-catenin**↓**	Yuanwen Chen et al.,2020 (32)
	Gastric Cancer	AGS		Apoptosis	Keap1/Nrf2**↑** - NEK2**↓** - HMOX1**↑**	Jianyong Wu et al.,2024 (35)
		AGS		Proliferation,Inhibition of Apoptosis	NEK2**↑** - PP1/AKT**↑**	Hao Wanet al.,2021 (34)
	Esophageal squamous cell carcinoma	ECA109, TE1, KYSE30, KYSE150, KYSE410, KYSE450,	BALB/c nude mice	Apoptosis	NEK2**↓** - TRIM21**↑**	Dong Guo et al.,2024 (37)
	Colon Cancer	SW480 SW620		Proliferation,Migration, Invasion	microRNA-128**↑** - NEK2**↑**	Yusuke Takahashi et al.,2014 (40)
		H1299		Proliferation,Migration, Invasion	NEK2↑ - Wnt/β-catenin↑	Christopher P Neal et al.,2014 (54)
		HCT116, HT29,SW480, DLD-1	BALB/c nude mice	Proliferation,Migration, Invasion	NEK2↑ - Wnt/β-catenin↑	Min Ji Ko et al.,2022 (52)
	Hepatocellular Carcinoma	HepG2、Huh7、SMMC		Proliferation,Migration, Invasion	NEK2**↑** - MAPK**↑**	Mei-Xia Zhang et al.,2016 (42)
		HepG2, Hep3B,HuH7, SMMC7721,HCCLM3 Snu387, Snu475		Proliferation,Migration, Invasion	NEK2↑ - Wnt/β-catenin↑	Shuang Lin et al.,2016 (41)
		MHCC97H, MHCC97L, SMMC-7721, HepG2, Huh7, BEL7402, HCCLM3		Proliferation,Migration, Invasion	NEK2↑ - Wnt/β-catenin↑	Zhang, Y. et al.,2018 (58)
	Pancreatic Cancer	KPC	C57BL/6 and Balb/c nude mice	Proliferation,Migration	NEK2**↑** - PD-L1**↑**	Xiaozhen Zhang et al.,2021 (43)
	Cholangiocarcinoma	HuCCT1, TFK1, HuH28, ETK1, RBE, IHGGK	nude mice	Proliferation,Migration		Toshio Kokuryo et al.,2007 (44)
	Ovarian Cancer	SKOV3		Proliferation	PCAT1**↑** - NEK2**↑ -**Wnt/β-catenin**↑**	X.-L. LIU et al.,2019 (46)
	Cervical Cancer	HeLa,SiHa, HEK293T	Balb/c mice	Proliferation,Migration, Invasion	NEK2↑ - Wnt/β-catenin↑	Tie Xu et al.,2020 (47)
NEK3	Breast Cancer	T47D		Migration, Invasion	NEK3**↑** - PRL**↑**	SL Miller et al., 2007 (65)
NEK4	Lung Cancer	A549	nude mice	Migration, Invasion	TGF-β/Smad**↑** - NEK4**↑ -** EMT**↑**	Nian-Hua Ding et al., 2018 (69)
	Colorectal Cancer	H1299	nude mice	Migration, Invasion	NEK4**↑ -** Survivin**↑**	Nian-Hua Ding et al., 2018 (69)
NEK5	Breast Cancer	MDA-MB-231, MDA-MB-468 and MCF-7	nude mice	Proliferation,Migration, Invasion,Inhibition of apoptosis	NEK5**↑** - Cyclin A2**↑**	Jing Pei et al., 2019 (128)
		MCF7	CID/Beige mice	Migration,		Margarite D. Matossian et al., 2021 (77)
	Nasopharyngeal carcinoma	NP69	nude mice	Migration, Invasion	miR-381-3p**↓** - NEK5**↑**	C-H Zhao et al., 2020 (78)
NEK6	Breast Cancer	MCF-7 and T47D		Proliferation,Migration,	NEK6**↑** - Ki67**↑**	Zhixian He et al.,2018 (81)
	Hepatocellular Carcinoma	HepG2, Li-7, Huh-7, BEL-7402	nude mice	Migration, Invasion		Hao Zhang et al.,2023 (79)
		Huh7		Proliferation	NEK6**↑** - Ki67**↑**	Xiaolei Cao et al.,2012 (82)
		huh7、HepG2、Hep3B PLC/PRF/5		Proliferation	NEK6 **↑** - cdc2/cyclin B**↑**	Biao Zhang et al.,2014 (84)
	Ovarian Cancer	SKOV3	nude mice	Proliferation,Migration	NEK6**↑** - FOXO3**↓** - C-MYC**↑**	Jingchun Liu et al., 2024 (90)
	Serous Ovarian cancer	A2780, Hey and SKOV-6		Inhibition of apoptosis	HIF1-α**↑** - NEK6**↑**	Marta De Donato et al., 2015 (129)
	Osteosarcoma	Saos-2, MG-63		Apoptosis	miR-26a-5p**↑** - NEK6**↓**	Min Zhu et al., 2023 (92)
	Colon Adenocarcinoma	SW480、SW620、HCT-8、HCT-116		Migration, Invasion,Inhibition of apoptosis	miR-323a-3p**↓** - NEK6**↑**	Zhongshi Hong et al.,2022 (93)
	Renal Cancer	786-O		Migration, Invasion,Inhibition of apoptosis	NEK6**↑** - miR-141-3p**↓**	Yifei Liu et al.,2022 (94)
	Thyroid Cancer	TPC-1、FTC-133、SW579、BCPAP K1	nude mice	Migration, Invasion,Inhibition of apoptosis	circ_NEK6**↑** - miR-370-3p/MYH9**↓**	Fukun Chen et al.,2021 (97)
NEK7	Squamous Cell Carcinoma of the Head and Neck	UD-SCCNH		Proliferation	WHSC1**↑** - NEK7**↑**	Vassiliki Saloura et al.,2015 (98)
	Retinoblastoma	Y79		Proliferation	NEK7**↑** - CKD2**↑**	Jian Zhang et al.,2018 (100)
	Hepatocellular Carcinoma	HepG2, Huh7,Hep3B, SMMC7721	nude mice	Migration, Proliferation	Nek7**↑** - Cyclin B1**↑**	Lei Zhou et al.,2016 (102)
		Hep3B,Huh7, HepG2, MHCC97L, MHCC97H,	nude mice	Inhibit pyroptosis	NEK7**↑** - IL-1β and LDH**↓**	Zilong Yan et al.,2022 (101)
	Pancreatic Cancer	AsPC-1, CFPAC-1, Capan-2, KP-2, SW1990, Panc-1, BxPC-3, KP-3, HPNE	nude mice	Proliferation,Migration, Invasion	NEK7**↑** - TPOCD110**↑**	Zilong Yan et al.,2021 (103)
	Gastric Cancer	MKN-45, MGC-803, HEK-293	mice	Proliferation,Migration, Invasion	NEK7**↑** - cGMP-PKG**↑**	Yi-Ke Li et al.,2021 (105)
NEK8	Breast Cancer	ing MDA-MB-231, BT549, HCC38	NSG mice	Proliferation,Migration, Invasion	NEK8**↑** - WNT/β-catenin**↑**	Eunji Kang et al.,2023 (131)
	Gastric Cancer	SGC-7901 SNU-1	BALB/C-nu/nu mice	Inhibition of Proliferation, Invasion,Migration	pVHL**↑** - NEK8**↓**	XIAO-FEI DING et al.,2018 (109)
NEK9	Breast Cancer	WHIM3 WHIM12	NU/J mice	Proliferation	NEK9**↑** - MAP2K4**↑**	Filip Mundt et al.,2018 (135)
	Gastric Cancer	AGS	nude mice	Proliferation, Invasion,Migration	SLIT2**↑** - ROBO1**↑** - NEK9**↑**	Guofang Lu et al.,2023 (117)
		AGS, MKN28 MKN45	BALB/C nude mice	Proliferation, Invasion,Migration	IL-6**↑** - STAT3**↑** - NEK9**↑**	Guofang Lu et al.,2021 (118)
	Colon Cancer	SW480 SW620		Proliferation, Invasion,Migration	NEK9**↑** - EG5**↑**	Meejeong Kim et al.,2023 (120)
	Pancreatic Cancer	PDAC	Pdx1-Cre and LSL-Trp53 R172H/+ mice	Proliferation, Invasion,Migration	NEK9**↑** - Hippo**↑**	Huizhen Nie et al.,2021 (133)
NEK10	Lung Adenocarcinoma	A549	NOD/SCID mice	Proliferation, Invasion,Migration	NEK10**↑** - β-catenin**↑**	Previn Dutt et al.,2024 (122)

**Table 3:** The potential therapeutic inhibitors of the NEK family members.

Kinases	Cancer type	Cell line	Animal	Agent	Molecular mechanism	References
NEK1	Postate Cancer	LNCaP cells	NOD-SCID mice	Thioridazine	Indirect inhibition through pNek1-pATRpC hk1 DDR pathway	Vibha Singh et al.,2019 (19)
	Glioblastoma	U87MG	Wistar rats	TMZ	TMZ indirectly enhances the effect of NKE1 inhibitors on glioblastoma	Luiza Steffens Reinhardt et al., 2022 (136)
NEK2	Lung Cancer	A549		Propynamide16	Directly inhibit NEK2	Jeffrey C. Henise et al., 2011 (137)
	Breast Cancer	MDA-MB-231 MDA-MB-468	nude mice	INH1	Directly inhibit NEK2	Chun-Mei Hu et al.,2015 (138)
NEK3	Breast Cancer	T-47D and MCF-7		U0126	U0126 inhibits MEK1/2- ERK1/2 signaling pathway, which leads to a significant reduction of phosphorylation of NEK3 at Thr-165 site, thus indirectly inhibiting the progress of breast cancer	Katherine M. Harringtont et al., 2016 (64)
NEK4	Lung Cancer	A549 cells	nu/nu mice	TRAIL	TRAIL can selectively mediate tumor cell death	So Jung Park et al., 2016 (68)
NEK6	Ovarian Cancer	SKOV3	nude mice	Paeonol	Paeonol directly inhibits NEK6 kinase activity and suppresses tumor growth	Jingchun Liu et al., 2024 (90)
NEK7	Colon Cancer	HCT116 HT29	BABL/c-nu mice	Quercetin	silencing NEK7 restrains the activation of the NLRP3 inflammasome-GSD-MD pathway, thus attenuating pyroptosis triggered by Que in colon cancer cells	Shi-Han Feng et al.,2024 (107)
NEK8	Hepatocellular Carcinoma	Huh-7 HepG2		esiRNA1	esiRNA1modulates HCC cell proliferation via direct downregulation of NEK8	Wen-Ling Chan et al.,2013 (110)
NEK9	Melanoma	1205Lu WM1366		Dabrafenib	Dalafini directly inhibits NEK9 and suppresses the proliferation and metastasis of melanoma	Manali Phadke et al.,2017 (119)

## 2 Structural characteristics of NEK family members and their role in the cell cycle

**Figure 2:**
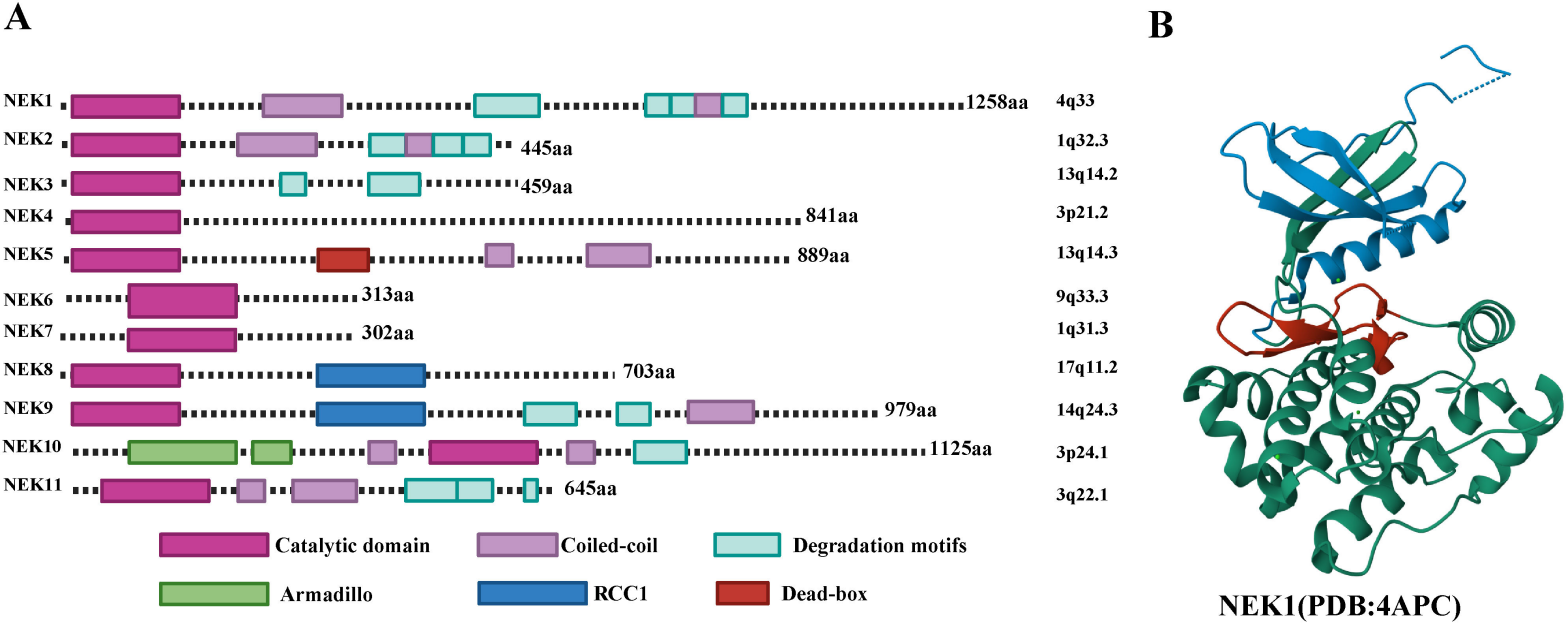
**(A)** Structural characteristics of the human NEK family. **(B)** Experimental crystal structure details of NEK1. Blue represents the N-terminal lobe, green represents the C-terminal lobe, and red represents the activation loop.

The kinase domain of NEK family proteins contains two subdomains: a small N-lobe and a large C-lobe (**Figure 2A**). The structural characteristics of the NEK family determine their diversity in cellular functions. Most NEK family members possess an N-terminal catalytic domain that contains a His-Arg-Asp (HRD) motif, and there is a serine/threonine residue in the activation loop which may be the site of activation modifications. In some NEK family members, this residue undergoes autophosphorylation, while in others, it is phosphorylated by an upstream kinase (9, 10). The C-terminal regions exhibit significant variation in length, sequence, and structure, and their diversified domains allow them to localize in different organelles and participate in specific cellular functions.NEK1 is the longest, possessing a typical N-terminal kinase domain capable of catalyzing the phosphorylation of serine/threonine residues; its C-end contains various functional domains, including multiple coiled-coil regions and domains that regulate protein-protein interactions. The experimental crystal structure details of NEK1 (PDB id: 4APC) are shown in **Figure 2B**.The C-terminus of NEK2 contains a coiled-coil domain. Such as: there are three splicing variants of NEK2 in mammals:NEK2A and NEK2B differ at their carboxy terminus, while NEK2C lacks the 8 amino acid sequence at the carboxy terminus of NEK2A. NEK2A is uniformly distributed in the nucleus and cytoplasm, NEK2B is mainly found in the cytoplasm, and NEK2C is primarily located in the nuclear region (11). NEK3 is relatively short and contains only a conserved N-terminal kinase domain. NEK5 has a unique DEAD-box domain, which is not present in other NEK family members (5). The sequence identity of the kinase regions of NEK6 and NEK7 exceeds 85%, both lacking complex C-terminal regulatory domains. Thus, their kinase activity mainly relies on interactions with other proteins (such as NEK9) (12). NEK9 has a long C-terminal structure that contains RCC1-like repeat sequences, allowing it to interact with NEK6 and NEK7 to regulate their activity. Generally, autophosphorylation typically occurs within the activation loop of the kinase domain, but the non-catalytic regions at the C-terminus of NEK8 and NEK9 can regulate their localization and activation through autophosphorylation (13, 14).The kinase domain of NEK10 is located in the middle of the protein rather than at the N-terminus.

Cell cycle dysregulation is a major cause of tumorigenesis, and the human NEK family is extensively involved in various stages of the cell cycle. In particular, it controls cell mitosis (7). During mitosis,NEK2,NEK6,NEK7,and NEK9 cooperate to regulate the formation of the bipolar spindle, chromatin condensation, nuclear envelope breakdown, and cytokinesis.NEK3 is associated with cell proliferation and signal transduction, while NEK1,NEK4,NEK5,NEK7,NEK8, NEK10,and NEK11 are all related to DNA damage response.NEK1,NEK8,and NEK4 play roles in cilia in post-mitotic cells (7).Therefore, overexpression of NEKs leads to chromosomal instability and aneuploidy in cancer cells. Moreover, Overexpression of NEKs activates several carcinogenic pathways, resulting in cancer development by acquiring proliferation, invasion, anti-apoptosis, and resistance capabilities.

### 2.1 Role of NEK Family Members in Malignant Tumors

#### 2.1.1 NEK1

In the NEK family,NEK1 is the gene most studied in relation to the occurrence and development of prostate cancer. In advanced prostate cancer, the level of phosphorylated NEK1 is significantly elevated. The Tousled Like kinases (TLK) is a phosphorylation inducer (15).TLK1 can activate NEK1 by phosphorylating the keto amino acid at position 141 (T141) of NEK1.Analysis of prostate cancer tissue samples reveals that the level of p-NEK1-T141 increases with higher Gleason scores (the grading system for prostate cancer),suggesting that this phosphorylated state may serve as a biomarker for disease progression (16).Early-stage prostate cancer typically relies on androgen signaling for growth, making androgen deprivation therapy (ADT) a common treatment strategy. VDAC1 is a channel protein on the outer mitochondrial membrane, and studies have found that TLK1 can form a complex with NEK1 to regulate the phosphorylation state of VDAC1,maintaining mitochondrial integrity and preventing apoptosis (17).NEK1 can also phosphorylate multiple residues on the key effector protein Yes-associated protein 1 (YAP1) in the Hippo pathway, enhancing the stability and nuclear retention of YAP1.Phosphorylated YAP1 can bind to the transcription factor transcriptional enhancer associated domain protein(TEAD)and the androgen receptor (AR),regulating gene expression and enhancing the growth and anti-apoptotic capacity of cancer cells, thereby promoting prostate cancer cell resistance to ADT (18).Many patients develop androgen-independent prostate cancer (AIPC) after treatment, which is no longer sensitive to conventional endocrine therapy, leading to treatment difficulties and poor prognosis. Research has shown that TLK1 and NEK1 are highly expressed in AIPC models, and inhibition of TLK1 or NEK1 expression disrupts DNA damage repair signaling pathways, making cells more sensitive to DNA damage. Inhibitors targeting the TLK1/NEK1 axis, such as Thioridazine, can effectively inhibit the growth of AIPC by modulating the activity of TLK1 without affecting its expression levels (19).The combination of Thioridazine with existing ADT can further enhance treatment efficacy, indicating its potential value as a combined therapy strategy.

Additionally, the high expression of NEK1 leads to the development of other tumors. For example, Yanbing Shen and colleagues (20) found that high expression of NEK1 is associated with poor prognosis of thyroid cancer. Talita Diniz Melo-Hanchuk and others further discovered that NEK1 is specifically overexpressed in follicular thyroid carcinoma, with higher levels found in patients with multifocal tumors and lymph node metastasis (6).Moreover,NEK1 expression is elevated in human glioma tissues and human glioma cell lines, correlating with lower survival rates in patients. Jun Zhu and colleagues found (21) that overexpression of NEK1 in glioma cells may be an important TMZ resistance factor. Silencing the NEK1 gene using siRNA significantly inhibited tumor cell growth and induced apoptosis, while also reduce resistance to temozolomide (21). Yumay Chen and colleagues (22) found increased expression of NEK1 in renal cancer. Compared to normal renal tubular epithelial cells, the expression of NEK1 was significantly increased in renal carcinoma tissues and cultured renal carcinoma cell lines. Overexpression of NEK1 results in cancer cells exhibiting greater resistance to DNA-damaging treatments, while downregulation of the NEK1 gene significantly enhances cancer cell sensitivity to these treatments and induces apoptosis. Guang Chen and others have confirmed through fundamental experiment that (23),the VHL protein is an important tumor suppressor and is often involved in the degradation of hypoxia-inducible factor (HIF) protein family. VHL may be involved in the regulation of NEK1. In renal cancer cells with VHL function loss, the stability of HIF-2α increases, and HIF-2α can directly bind to the hypoxia-responsive element (HRE) in the NEK1 gene promoter, thereby enhancing NEK1 expression. This suggests that NEK1 may be a new hypoxia-inducible gene (24).

#### 2.1.2 NEK2

NEK2 is the most extensively studied gene among various types of cancer.NEK2 expression is low in the G1 phase of the cell cycle, increases in the S and G2 phases with enhanced activity, peaks in the late G2/M phase, and decreases as the cell enters mitosis.Additionally,NEK2 is responsible for initiating centrosome separation during the G2/M phase of the cell cycle (25).Overexpression of NEK2 leads to chromosomal instability and aneuploidy in cancer cells (26).NEK2 is expressed at higher levels in many human cancers compared to normal tissues, including prostate cancer (27),renal cancer (28, 29),non-small cell lung cancer (30, 31),breast cancer (32, 33),gastric cancer (34, 35),esophageal cancer (36, 37),colorectal cancer (38–40),hepatocellular carcinoma (41, 42),pancreatic cancer (43),cholangiocarcinoma (44),ovarian cancer (45, 46),cervical cancer (47),oral squamous cell carcinoma (48),and malignant peripheral nerve sheath tumors (49).Thus, elevated NEK2 expression appears to be a common feature in most human tumors. Studies report that NEK2 expression influences cancer cell proliferation (50, 51).NEK2’s pro-proliferative role may be related to the Wnt/β-catenin pathway. NEK2 can phosphorylate β-catenin, promoting its nuclear translocation (52–54), which the expression of Wnt target genes is activated and cell proliferation is affected.Furthermore,NEK2 can inhibit apoptosis and promote cell survival (31). The mechanism by which NEK2 inhibition leads to apoptosis in tumor cells is still unclear. Hayward and colleagues reported for the first time that overexpression of NEK2 precedes cancer cell metastasis (55).NEK2 is believed to participate in invasion and motility, and promote cancer metastasis (56, 57). NEK2’s pro-metastatic role may also be related to the Wnt/β-catenin pathway. Research shows NEK2 overexpression induces nuclear accumulation of β-catenin in lung cancer cells (26). Additionally, NEK2 can enhance tumor cell invasion and metastasis by regulating the epithelial-mesenchymal transition (EMT) process (58). Drug sensitivity and resistance are important issues in cancer treatment. Some researchers have reported that NEK2 is involved in resistance to 5-fluorouracil,tamoxifen, trastuzumab, paclitaxel, and doxorubicin (59–61).NEK2 and Plk4 may promote the proliferation of cancer cells with genomic alterations, thereby affecting the uptake, metabolism, and cellular response to therapeutic drugs, leading to drug resistance (60).Meanwhile, NEK2 gene silencing combined with paclitaxel or doxorubicin can enhance the sensitivity of TNBC cells to chemotherapy (59).

#### 2.1.3 NEK3

Unlike NEK1, NEK3 is primarily overexpressed in papillary thyroid carcinoma with high cellularity. Quantitative analysis of NEK3 expression can distinguish between benign and malignant thyroid tissues, with a sensitivity of 78%, specificity of 80%, and accuracy of 79%.This suggests that NEK3 expression is closely related to the malignancy and invasiveness of thyroid cancer (6).According to the literature, NEK3 expression is upregulated in esophageal adenocarcinoma and its precursor lesion, Barrett’s esophagus (62).NEK3 is overexpressed in gastric cancer and is significantly associated with TNM staging, lymph node metastasis, and poor prognosis (63).In a breast cancer cell models, NEK3 phosphorylation site Thr165 is a key regulator of cancer cell migration and local adhesion remodeling (64).The phosphorylation of NEK3 Thr-165 depends on the activation of the MEK1-ERK1/2 signaling pathway in response to Prolactin (PRL) stimulation in vivo.U0126 can inhibit PRL induced activation of the MEK1-ERK1/2 signaling pathway, thereby significantly reducing the phosphorylation level of NEK3 at the Thr-165 site (64). PRL, which plays a critical role not only in mammary gland development and milk production but also as a pro-carcinogenic factor in breast cancer progression and metastasis, has been shown by SL Miller and colleagues to promote breast cancer cell motility by activating NEK3.This activation leads to changes in the phosphorylation state of cytoskeletal proteins, such as Cofilin, triggering cytoskeletal reorganization and enhancing cell movement (65).

#### 2.1.4 NEK4

Currently, research on NEK4 in cancer is limited, but it has been reported to be involved in mediating cancer cell death pathways. Although apoptosis can inhibit tumor development and metastasis, cancer cells often acquire the ability to overcome these barriers. The tumor necrosis factor-related apoptosis inducing ligan(TRAIL), a member of the tumor necrosis factor(TNF)family,can trigger apoptosis. Since TRAIL receptors are primarily expressed on cancer cells, TRAIL can selectively mediate tumor cell death. TRAIL has been considered a potential cancer therapeutic agent (66, 67).Survivin is an anti-apoptotic protein which highly expressed in most cancers and leads to chemotherapy resistance, increased tumor recurrence and shortened survival. In vitro studies have found that the loss of NEK4 increases the sensitivity of colorectal and lung cancer cells to down-regulate Survivin-mediated TRAIL-induced apoptosis (68).Lung cancer is a highly metastatic malignant tumor, and epithelial-mesenchymal transition (EMT) is a key process for tumor cells to acquire the ability to invade and metastasize. Both in vivo and in vitro studies have confirmed that NEK4 kinase promotes EMT in lung cancer cells, enhancing their migration and invasion abilities, thereby promoting lung cancer metastasis (69).Suppressing NEK4 increased E-cadherin and Zo1 (epithelial markers), decreased Zeb1 and Smads (mesenchymal markers), and reduced metastasis in mice. This suggests NEK4 enhances EMT by downregulating epithelial markers and upregulating mesenchymal transcription factors, likely through the transforming growth factor-β (TGF-β)/Smad pathway (69).Quercetin is a flavonoid and has diverse biological effects. Actually it is well defied that quercetin can act on the chemo-sensitization and radio-sensitization in various cancer cells (70, 71).Quercetin inhibits protein kinases involved in deregulated cell growth in cancer cells, and NEK4 is a known target of quercetin (72).

#### 2.1.5 NEK5

Although research on NEK5’s mechanisms in cancer is limited, it can regulate the integrity of cell centrosomes, sequencing data has highlighted its potential as a new target in the cancer field. For instance, microarray analysis has shown that NEK5 expression is upregulated in highly metastatic non-small cell lung cancer (NSCLC) cells compared to low-metastatic cell lines (73).In 2017,RNA sequencing of prostate cancer tissue samples identified NEK5 as a novel RNA candidate biomarker for the disease (74).In osteosarcoma, information from the PHAROS database suggested that NEK5 could be a potential target (75).While these studies link NEK5 expression or signaling with various solid tumor subtypes, all of the data is based on bioinformatics analysis, without further experimental validation. The exact mechanism by which NEK5 drives tumorigenesis remains unclear. In breast cancer, clinical evidence has shown that high NEK5 expression is significantly associated with tumor progression and poor prognosis. Inhibition of NEK5 expression was found to effectively inhibit the proliferation, migration and invasion of tumor cells both in vitro and in vivo. Some mechanism studies suggest that NEK5 upregulates Cyclin A2 expression while downregulating Cyclin D1, Cyclin D3, and Cyclin E1 (76).Another study confirmed that NEK5 activation promotes EMT in breast cancer, thereby enhancing cell migration and invasion (77).In nasopharyngeal carcinoma,NEK5-mediated miRNA regulation has been shown to inhibit hypoxia-induced glycolysis, migration, and invasion. NEK5 is a direct target of miR-381-3p,and interfering with NEK5 can mitigate the pro-glycolytic and pro-metastatic effects of miR-381-3p downregulation (78).

#### 2.1.6 NEK6

The loss of NEK6 function can lead to mitotic abnormalities, metaphase cell accumulation, chromosome segregation errors, and subsequent apoptosis, involving in the progression of the mitotic cell cycle (79). Recent years, research has found NEK6 is linked to the development of many tumors, with significantly elevated expression levels in solid tumors (79). Bioinformatics analysis suggests that NEK6 is highly expressed in breast cancer and is associated with reduced survival rates in breast cancer patients (80). In breast cancer, both in vivo and in vitro studies have shown NEK6 overexpression promotes proliferation and migration in cancer cells (81). NEK6 expression in hepatocellular carcinoma (HCC) tissue is significantly higher than in normal liver tissue. High NEK6 expression is associated with tumor grading, staging, and poor prognosis (82, 83).NEK6 levels in liver cancer cell lines such as Huh7, Hep3B,and HepG2 are also elevated. Overexpression or inhibition of NEK6 can significantly impact the proliferation of liver cancer cells. Biao Zhang et al. pointed out that NEK6 regulates cyclin B transcription by activating CDC2, promoting the progression of the G2/M phase of the cell cycle. Thereby promoting tumor proliferation (84).In liver cancer,NEK6 inhibits TGF β - mediated transcriptional activity by interacting with Smad4, thereby affecting cell growth and differentiation. Specifically, overexpression of NEK6 inhibits TGF β - induced cell growth arrest and promotes hepatoma carcinoma cell proliferation. Research has found that NEK6 can promote hepatoma carcinoma cell proliferation by regulating the expression of cell cycle related proteins such as Cyclin B and CDC2. NEK6 is also associated with the invasion and metastasis ability of hepatoma carcinoma, and it may affect the migration and invasion ability of hepatoma carcinoma cells by regulating the reorganization of the cytoskeleton and related signaling pathways (85). Additionally, high NEK6 expression in gastric cancer and head and neck squamous cell carcinoma is positively correlated with distant metastasis, lymph node metastasis, TNM staging, as well as poor prognosis. Downregulation of NEK6 expression can inhibit cancer cell invasion and migration (86, 87).Kasap and colleagues reported (88) that NEK6 expression in adenomatous polyps and colorectal adenocarcinoma tissue is significantly higher than in normal colorectal mucosa. Another study found that NEK6 expression in ulcerative colitis and colorectal cancer tissue is significantly elevated compared to normal colorectal mucosa, with the top levels observed in colorectal cancer tissue (89). This suggests that NEK6 plays a role in the early stages of intestinal inflammation, especially in patients with extensive colonic involvement and long-term ulcerative colitis. NEK6 is also involved in prostate cancer, where its knockdown increases intracellular redox balance, enhances DNA damage response, induces apoptosis, and increases sensitivity to chemotherapy drugs. Further studies have revealed that NEK6 enhances androgen receptor signaling, maintaining cell growth in a castration-resistant environment, thus promoting castration resistance. NEK6 is likely a key target for castration-resistant prostate cancer. In ovarian cancer, NEK6 promotes chemotherapy resistance by affecting the interaction between FOXO3 and C-MYC (90).Another study found that NEK6,together with HIF-1α,co-regulates the cytoskeleton, affecting chemotherapy resistance in serous ovarian cancer (91).Quercetin can dose dependently reduce the expression of NEK6 in HeLa cells and has tumor suppressive effects. S206A is a potential NEK6 inhibitor. These two drugs can be used as cancer treatments.

Circular RNAs (circRNAs) and microRNAs (miRNAs) are closely related to the occurrence and progression of various types of cancer through their abnormal expression. Numerous studies have shown that NEK6 influences the development of osteosarcoma (92), colorectal cancer (93), kidney cancer (94), and retinoblastoma (95) by interacting with miR-26a-5p, miR-323a-3p, miR-141-3p, and miR-506-3p. Circ_NEK6 is typically transcribed from the exons of the NEK6 gene. Overexpression of NEK6 circRNA can promote the growth and invasion of thyroid cancer cells (96), while also increasing the resistance of differentiated thyroid cancer cells to iodine-131 (¹³¹I) therapy (97).

#### 2.1.7 NEK7

NEK7 is one of the smallest proteins in the mammalian NEK family. For the past few years, many research have reported which the upregulation of NEK7 is linked to the development of several cancers, including head and neck squamous cell carcinoma (98), gallbladder cancer (99), retinoblastoma (100), liver cancer (101, 102), pancreatic cancer (103), lung cancer (104), and esophageal cancer (62), indicating its potential as a biomarker. In gastric cancer, NEK7 is overexpressed and associated with poor prognosis. Cell experiments have shown that NEK7 promotes cell proliferation, while the lack of NEK7 leads to inhibited gastric cancer proliferation and G1/S phase arrest.NEK7 not only directly regulates cancer cell proliferation but may also promote the progression of gastric cancer by interacting with other cells, influencing immune infiltration and changes in immune cell subpopulations (105). In recent years, more and more studies have highlighted NEK7 as an important regulatory factor, playing a key role in the inflammatory response by promoting the assembly and activation of the NLRP3 inflammasome, leading to the release of inflammatory cytokines such as IL-1β and IL-18. The activation of NLRP3 inflammasome mediated by NEK7 is closely related to a variety of inflammatory diseases, including autoimmune, metabolic, and infectious diseases (106).Preliminary results from NEK7 inhibitors have shown promising anti-tumor and anti-inflammatory effects. Quercetin induces pyroptosis in colon cancer cells by activating the NEK7-mediated NLRP3 inflammasome-GSDMD signaling pathway, suggesting quercetin’s potential in colon cancer treatment and providing a foundation for future research (107).

#### 2.1.8 NEK8

Few studies have reported a connection between NEK8 and tumor development. Most NEK8-related research focuses on its association with cystic kidney disease. However, in recent years, there have been reports indicating that NEK8 plays a pro-cancer role in breast cancer (108), gastric cancer (109), liver cancer (110), glioma (111) and colon cancer (112, 113). The exact mechanism by which NEK8 promotes cancer is not fully understood. Some studies suggest that NEK8 may potentially exert its pro-cancer effect in human gastric cancer through the HIF-1 signaling pathway (109). Von Hippel-Lindau protein(pVHL) interacts with NEK8 and promotes its degradation. Since hypoxia stabilizes HIF-α by preventing its degradation by pVHL, it can be inferred that in a hypoxic tumor environment, HIF-α levels would increase. As NEK8 is regulated by HIF-α (114), this would likely lead to an increase in NEK8 expression. Additionally, the Kaplan - Meier survival analysis (109) that higher expression levels of NEK8 are associated with poor survival in gastric cancer patients. Considering that tumor hypoxia is often related to poor prognosis, it is reasonable to suspect that tumor hypoxia may contribute to increased NEK8 expression, which in turn may promote tumor progression. Knocking down NEK8 can inhibit cell proliferation (110), induce cell cycle arrest, suppress cell migration and invasion, and also reduce the characteristics of tumor stem cells. The WNT/β-catenin pathway plays a crucial role in various cellular processes, including cell growth, differentiation, tumorigenesis, chemoresistance, and cell cycle progression. β-catenin translocation into the nucleus triggers a range of transcription factors, regulating downstream signaling cascades. Eunji Kang and colleagues (108) reported that in breast cancer, NEK8 overexpression promotes β-catenin nuclear translocation, enhancing its activity within the nucleus. Additionally, NEK8 overexpression may induce the phosphorylation of proteins related to cancer progression. Furthermore, co-immunoprecipitation results showed that NEK8 directly interacts with β-catenin, suggesting that NEK8 is responsible for the phosphorylation of β-catenin at Ser552 and Ser675. This phosphorylation leads to the stabilization and nuclear accumulation of β-catenin.

#### 2.1.9 NEK9

The clinical research on NEK9 in breast cancer is controversial. Zhu and colleagues (115) found that NEK9 expression was elevated in tissue samples from breast cancer patients and was positively correlated with the occurrence and development of breast cancer, serving as an independent risk factor for poor prognosis. However, Xu and colleagues (116) reached the opposite conclusion: they discovered that NEK9 expression was downregulated in breast cancer and that low NEK9 expression was associated with larger, poorly differentiated tumors, lymph node metastasis, distant metastasis, and HER2-positive tumors. Patients with low NEK9 expression had significantly lower overall survival (OS) and disease-free survival (DFS) compared to those with high NEK9 expression. Gao and colleagues (80), on the other hand, found that compared to normal breast tissue, NEK9 was highly expressed in breast cancer tissues, and its high expression was positively correlated with disease-free survival in breast cancer patients. Currently, the role of NEK9 in breast cancer lacks experimental validation. NEK9 has a pro-cancer role in gastric cancer. In gastrointestinal tumors, NEK9 expression is elevated in metastatic gastric cancer lesions compared to non-cancerous tissue and primary cancer lesions, and its increased levels are associated with reduced overall survival in gastric cancer patients (117). Further research revealed that cancer-associated fibroblast-secreted SLIT2 could specifically activate NEK9, significantly promoting gastric cancer cell migration and invasion (117). Additionally, NEK9 acts as an effector of the IL-6/STAT3 signaling pathway. The IL-6/STAT3 pathway can upregulate NEK9 expression, thereby targeting the phosphorylation of ARHGEF2, promote the ability of gastric cancer cell migration and metastasis (118).Dabrafenib can inhibit NEK9, and in cancer cell lines with NRAS - and KRAS - mutations, dabrafenib has a significant inhibitory effect on cancer cell growth by inhibiting NEK9 and CDK16. This suggests that dabrafenib or its analogues may be developed as inhibitors against NEK9 for the treatment of related cancers (119).In colorectal cancer, analysis of clinical data shows that high expression of NEK9 and EG5 is associated with poor prognosis in patients with pathological stage t3 colon cancer. Intervening in the NEK9-EG5 axis could regulate the proliferation, migration, and invasion abilities of colorectal cancer cells (120).Pancreatic cancer is a highly malignant cancer type with a very poor prognosis, and patients have a very low 5-year survival rate. The prolactin receptor (PRLR) belongs to the cell membrane receptor family and primarily mediates the biological effects of prolactin. PRLR exists in two main isoforms: the long isoform (PRLR-LF) and the short isoform (PRLR-SF). PRLR-SF, as an significant metabolic suppressor in pancreatic cancer, can interact with NEK9. The loss of PRLR-SF can activate NEK9, thereby inhibiting the phosphorylation of YAP, a key tumor suppressor in the Hippo pathway. This inhibition leads to enhanced nuclear translocation and transcriptional activity of YAP, which promotes the proliferation and growth of pancreatic cancer cells. Inhibiting the NEK9-Hippo axis activities could be a crucial breakthrough in the treatment of pancreatic cancer.

#### 2.1.11 NEK10

There is little research on the function of NEK10, except for its involvement in cell cycle regulation. Wen-Liang Gao and colleagues (121) conducted bioinformatics analysis and found that NEK10 is expressed at higher levels in normal breast tissue compared to breast cancer tissue, and that Low expression of NEK10 can lead to poor prognosis in breast cancer patients, Indicating its potential tumor suppressive function. However, this study lacks experimental validation. In contrast, NEK10 acts as an oncogene in lung cancer. NEK10-mediated tyrosine phosphorylation enhances the stability of β-catenin, reducing its chances of ubiquitination and degradation. When NEK10 is overexpressed, lung cancer cells exhibit stronger proliferation and migration capabilities. NEK10, as a potential tumor-promoting factor, could serve as a new therapeutic target for lung adenocarcinoma (122).

#### 2.1.12 NEK11

NEK11 exhibits both oncogenic and tumor-suppressive functions across different cancer types. NEK11 is necessary for HCT116 cells to produce G2/M phase blockade against ionizing radiation (IR) or chemotherapy drug irinotecan. When NEK11 is depleted by RNAi mediation, cells are unable to enter the G2/M phase arrest normally after DNA damage, but continue to enter the cell cycle, which may cause cells to divide with damaged DNA, leading to genomic instability (123).A research team conducting whole-genome sequencing on melanoma families discovered multiple mutations in NEK11 among melanoma patients, which could lead to its dysfunction. Functional experiments and cell models revealed that cells with NEK11 mutations show increased proliferation and anti-apoptotic properties, suggesting NEK11’s potential role in melanoma development. The link between NEK11 and colorectal cancer has also been identified (124). Studies have shown that the loss of NEK11 in colorectal cancer cells prevents effective DNA repair after damage, leading to cell death. Targeting NEK11 may enhance the efficacy of DNA-damaging agents in treatment (123).

Research has also indicated high NEK11 expression in breast cancer, which is associated with better prognosis (80). Ovarian cancer, a highly lethal gynecological tumor, often develops resistance to chemotherapy, leading to treatment failure. In a 2014 study by Liu et al. (45), the relationship between NEK11 and ovarian cancer was validated. The study concluded that NEK11 expression is significantly lower in chemoresistant ovarian cancer tissues compared to chemosensitive tissues, and NEK11 downregulation is associated with poor prognosis and high recurrence rates. Overexpression of NEK11 can significantly enhance the sensitivity of ovarian cancer cells to chemotherapy, inducing apoptosis. Bioinformatics analysis revealed that NEK11 mRNA is significantly downregulated in ovarian cancer tissues and cisplatin resistant cells (125).NEK11 may serve as a novel biomarker for chemoresistance in ovarian cancer and as a potential therapeutic target, offering new strategies to overcome chemotherapy resistance in ovarian cancer.

## 3 Summary

The abnormal expression of NEK family genes is involved in the initiation, maintenance, progression, and metastasis of various cancers. These findings indicate that NEKs could be a potential therapeutic target for many cancer treatments. The structural diversity of the NEK family makes them promising drug targets in cancer research. Inhibitors designed to target specific domains, such as kinase domains or protein-binding domains, may offer high selectivity, effectively interfering with cancer cell proliferation and migration. More research is needed to develop targeted interventions for NEKs, which could become an important strategy for future cancer therapies.
